# How do external factors contribute to the hypocoagulative state in trauma-induced coagulopathy? – In vitro analysis of the lethal triad in trauma

**DOI:** 10.1186/s13049-018-0536-8

**Published:** 2018-08-15

**Authors:** Michael Caspers, Nadine Schäfer, Matthias Fröhlich, Ursula Bauerfeind, Bertil Bouillon, Manuel Mutschler, Marc Maegele

**Affiliations:** 1 The Institute for Research in Operative Medicine, Faculty of Health, Department of Medicine, Witten/Herdecke University, Ostmerheimer Str. 200, 51109 Cologne, Germany; 2 Department of Traumatology, Orthopaedic Surgery and Sports Traumatology, Cologne-Merheim Medical Centre (CMMC), Witten/Herdecke University, Campus Cologne-Merheim, Ostmerheimer Str. 200, 51109 Cologne, Germany; 3 Department of Transfusion Medicine, Cologne-Merheim Medical Centre (CMMC), Witten/Herdecke University, Campus Cologne- Merheim, Ostmerheimer Str. 200, 51109 Cologne, Germany

**Keywords:** Lethal triad, Hypothermia in trauma, Acidosis in trauma, Dilution in trauma, ROTEM^®^, PlateletMapping^®^

## Abstract

**Background:**

External factors following trauma and iatrogenic intervention influence blood coagulation and particularly clot formation. In particular, three external factors (in detail dilution via uncritical volume replacement, acidosis and hypothermia), in combination, referred to as the “lethal triad”, substantially aggravate the hypocoagulative state after trauma. Contribution of these external factors to the resulting hypocoagulative state in trauma and especially their influence on primary haemostasis has still not been investigated systematically.

This study aims to assess this contribution to the aggravating hypocoagulative state in trauma-induced coagulopathy (TIC) using an in vitro simulation assay. Emphasis is given to platelet contribution to clot formation and to the investigation of how platelet activation alters under the respective conditions.

**Methods:**

To simulate the conditions of lethal triad in vitro, whole blood samples taken from five healthy volunteers were introduced to the respective conditions. Besides standard coagulation testing, thrombelastometric analysis and differentiated platelet mapping were performed.

**Results:**

All three simulated conditions induced significant impairments of clot formation (clot formation time, CFT; α -angle) and propagation (maximum clot firmness, MCF; Diameter A5-A25), with the highest impact under hypothermia and dilution. Consistently, lethal triad resulted in an additive effect of all conditions. None of the simulated conditions induced a statistically relevant change in coagulation initiation assessed by EXTEM and FIBTEM thrombelastometry.

Platelet contribution to clot formation decreased gradually under the respective conditions, reaching statistical significance for simulated dilution, and attaining its greatest extent under the conditions of lethal triad (Δtrias/baseline 0.59; *p* = 0.01). Consistent, reduced CD62 expression levels were observed under experimental acidosis (Δacidosis/baseline 0.32; *p* = 0.006), dilution (Δdilution/baseline 0.34; *p* = 0.01) and lethal triad (Δlethal triad/baseline 0.24; *p* = 0.01).

**Conclusion:**

The respective external factors of lethal triad play a pivotal role in the development of coagulopathy, essentially influencing the kinetics of clot formation, and to a varying extent clot diameter, as measured by thrombelastometry. Moreover, impairment of platelet function under the conditions of lethal triad plays a key role in the pathophysiology of TIC, resulting in reduced responsiveness to stimulation with ADP that might also be present after trauma. Our data indicate that impairment of primary haemostasis contribute to the hypocoagulative state in TIC after trauma aggravated by external factors of lethal triad.

## Background

Haemorrhage after major traumatic injury is still the major global cause of lost life years [1–4]. On admission, one-quarter of trauma patients suffer from a “trauma-induced coagulopathy (TIC)” associated with massive blood loss and a higher incidence of multiple organ dysfunction, resulting in fourfold higher mortality [5, 6]. Despite a profound upregulation in procoagulant mechanisms shortly after trauma, the main triggers for TIC have been identified as hypoperfusion, including hyperfibrinolysis, endothelial activation and platelet dysfunction, resulting in a primary and independent entity [5, 7–9]. In the further sequalae, this entity is substantially aggravated by iatrogenic interventions such as uncritical volume resuscitation leading to haemostatic depletion via dilution, acidosis and hypothermia, which, in combination, are referred to as the “lethal triad” [10]. Each condition contributes to a vicious and progressively worsening cycle, aggravating coagulopathy and resulting in a severely deleterious state for the trauma patient.

In contrast, coagulation analysis in trauma based on standard laboratory assays is hindered due to its methodological composition, as this does not reflect lethal triad conditions [11]. In addition, the validity of abnormalities in laboratory-based coagulation testing and their significance to a clinically relevant bleeding phenotype is still unclear as there is still an insufficient evidence that abnormal coagulation test results predict reliable a bleeding phenotype [12].

For viscoelastic assays, such as thromboelastometry, it could be shown that they enable a rapid evaluation of the patients’ coagulation status after hospital admission and can therefore be used as point-of-care (POC) technology. Thromboelastometry provides information about primary haemostasis and enzyme function after trauma, and its use is therefore recommended by current guidelines [13, 14]. But even though this technique has the potential to reflect the influence of external conditions on coagulation function, neither the contribution to coagulopathy of respective lethal triad factors in trauma, nor their combination, nor how they alter thrombelastometric testing have been investigated systematically.

In the present study, we aim to characterize the role of selected external factors reflecting the lethal clinical triad on clot initiation, dynamics and stability by using in vitro simulations. This approach allows the assessment of influences contributing to the lethal triad from different perspectives under standardized conditions, independent of physiological responses initiated rapidly to tissue injury and shock. The aim is to investigate both the influence of the respective condition on different phases of coagulation (plasmatic or platelet-based), and their combined affects. An emphasis has been given to the platelet contribution to clot formation and to the investigation of how platelet activation is altered under the respective conditions.

## Methods

### Volunteers and collection of whole blood samples

After the approval of the ethical committee of the University of Witten/Herdecke and in accordance with the Declaration of Helsinki, six healthy volunteers gave their written and informed consent to participating in this study. None of the donors had a medical history with respect to coagulopathy, a known bleeding disorder, or a treatment with any medication in the 10 days prior to the donation. One volunteer was excluded due to a pre-existing reduced platelet count (< 200.000/μl) along with a reduced haematocrit. A whole blood sample of 450 ml was then taken from each volunteer using a commercially available system containing 63 ml CPD (citrate-phosphate-dextrose-derivate) and 100 ml mannitol for conservation and coagulation inhibition (composelect^®^4F T&B-63 CPD/100 ml SAG-M-WB + PDS-M, ChBNo:41IB14FA00, Fresenius Kabi Deutschland GmbH, Bad Homburg, Germany; www.fresenius-kabi.de). To standardize the initial pH (median 7.35, IQR 0.02), sodium hydrogen carbonate (Sodiumhydrogencarbonat 8.4%- B. Braun Infusionslösung^®^, B.Braun Melsungen AG, Melsungen, Germany; www.bbraun.de) was added to the whole blood sample. After donation, samples were immediately aliquoted and introduced to the in vitro testing system using different sample tubes for the respective testing system (*Blood count:* 2,7 ml EDTA (1.6 mg EDTA/ml), S-Monovette^®^, Sarstedt, Germany; Standard Coagulation/ROTEM^®/^ROTEG^®^: 3 ml 9NC (9NC: 0.106 mol/l, S-Monovette^®^, Sarstedt, Germany; BGA: 2 ml LH (50 I.E. Heparin/ml Blood), S-Monovette^®^, Sarstedt, Germany.

### Lethal triad in vitro model

In order to simulate conditions of lethal triad, the whole blood sample was separated into five equal vials that were kept at 37 °C in constant movement. Within all samples, temperature and pH were measured on a continuous basis (pH 3310, WTW GmbH, Weilheim, Germany, Serial No. 15340435). Acting as a baseline sample, vial 1 did not undergo any further treatment. Conditions of lethal triad were chosen based on clinical reports indicating coagulopathy introduced by lowering pH < 7.0 [15], dilution up to 33% [16] and hypothermia to 32 °C [17]. Introducing the respective conditions, pH was lowered to pH 6.8 ± 0.05 using 2 M HCl by titration (vial 2, “acidosis”), the blood samples were diluted to 33% using crystalloids (vial 3, “dilution”; Sterofundin^®^ ISO Infusionslösung, B. Braun Melsungen AG, Melsungen, Germany, www.bbraun.de) or temperature was lowered to 32 °C (vial 4, “hypothermia”). Vial 5 (lethal triad) combined all three external factors by lowering the temperature to 32 °C and pH to 6.8 and diluting the sample to 33%. For excluding haemolysis, we measured free haemoglobin and the electrolytes Na^+^, K^+^, Ca^2+^ and Cl^−^ by blood gas analysis (BGA, GEM 3500, Serial No. 14074200) for each sample. Following this, a differentiated coagulation testing including Activated Partial Thromboplastin Time (aPTT) [s], Thrombin Time [s], Quick [%], INR [dimensionless], and fibrinogen [mg/dl] (via Clauss method), a standard blood count with haemoglobin [g/dl], platelets count [/μl], haematocrit [%], a ROTEM^®^ analysis, a ROTEG platelet Mapping^®^, and a platelet function testing was performed.

### ROTEM^®^ analysis

Rotational thrombelastometry (ROTEM^®^, Tem International GmbH, Munich, Germany) was used according to the manufacturer’s recommendations. Citrated blood samples were recalcified by adding 20 μl of CaCl_2_ and introduced to EXTEM and FIBTEM assay. Data were collected for the following variables: coagulation time (CT), clot formation time (CFT), α-angle, maximum clot firmness (MCF) and clot firmness after 5 (A5), 15 (A15) and 25 min (A25).

### PlateletMapping

Thromboelastography analysis was performed with the ROTEG™ Coagulation Analyzer (Haemonetics, Munich, Germany; www.haemonetics.de) using an ADP-PlateletMapping Assay according to the manufacturer’s instructions. Platelet aggregation in response to ADP was calculated on the basis of the formula: % aggregation = [(MA_ADP_ − MA_fibrin_)/(MA_thrombin_ − MA_fibrin_)] × 100.

### Platelet function testing

For platelet function testing, we used a kit provided by PlateletSolutions^®^ (Nottingham, UK, www.plateletsolutions.co.uk/products). Whole blood samples collected into sodium citrate tubes were introduced to the respective conditions of lethal triad. Samples were incubated for 5 min without mixing at the respective temperature and then fixed using PAMFix (Platelet Solutions Ltd., Nottingham, UK). For platelet stimulation, blood samples were treated with saline to provide a baseline test (baseline), ADP supplemented with U46619, (ADP/U4 10 μM and 1 μM respectively ADP) or with 20 μM thrombin receptor activating peptide (TRAP). Samples were then routinely analysed within 7 days by measuring expression of CD62 on thrombocytes using a flow-cytometric assay.

### Statistical analysis

Descriptive results are presented as median and interquartile range (IQR). Nonparametric Kruskal-Wallis test was performed to compare changes in laboratory data when comparing respective condition vs. baseline. All aggregated values are presented in mean and standard deviation and multiple students’ t-tests were performed when comparing condition vs. baseline or lethal triad vs. baseline. To test for normal distribution, the Shapiro-Wilk-test was performed. Results are described as “statistically significant” if their error probability was less than 5% (*p* < 0.05).

Statistical analyses were performed using GraphPad Prism version 7.00 for Windows (GraphPad Software, La Jolla California USA).

## Results

### Standard coagulation assays and blood count

Untreated blood samples obtained at baseline and assessed by using standard coagulation assays and blood count revealed values within physiological reference ranges as shown in Table 1. The exposure of blood samples to experimental acidosis and hypothermia was not associated with any significant changes in either standard coagulation assays or blood count. However, when blood samples were exposed to experimental 33% dilution, haemoglobin, haematocrit, platelets and fibrinogen concentration dropped by approximately one third, with a corresponding change in standard coagulation assays by 20–30%. The exposure to the combination of all three conditions (“lethal triad”) was associated with a further drop in platelets along with a slight further increase in prothrombin time (Table 1).

**Table 1:** Standard blood count and cougulation testing for the respective lethal triad conditions

	Blood count			Standard coagulation tests				BGA
	Haemoglobin level [g/dl]	Platelets [10^3/μl]	Haematocrit [%]	aPTT [s]	TT [s]	INR	Fibrinogen [mg/dl]	pH
Baseline	11.4 (0.3)	257 (103)	32.7 (2.5)	30.3 (0.3)	16 (0.3)	1.18 (0.1)	250 (59)	7.35 (0.02)
pH 6.8	12.6 (1.4)	211 (70)	35.4 (3.1)	27.5 (0.3)	17.5 (0.3)	1.14 (0.09)	268 (39)	6.83 (0.05)
Temp. 33 °C	11.4 (1)	247 (98)	33.1 (2.3)	32.1 (0.1)	15.7 (0.1)	1.16 (0.13)	243 (62)	7.38 (0.00)
Dilution	7.5 (0.9)	167 (44)	22.2 (2.3)	43.3 (0.6)	20.8 (0.6)	1.41 (0.07)	166 (39)	7.37 (0.02)
Lethal Triad	7.7 (0.8)	125 (59)	22.8 (2)	34.9 (0.8)	23.8 (0.8)	1.46 (0.11)	154 (27)	6.83 (0.02)

*Left column*: Results from standard blood count for the respective conditions, including haemoglobin level [g/dl], platelet count [/μl] and haematocrit [%]. *Right column:* Results from standard coagulation testing, including Activated Partial Thromboplastin Time (aPTT) [s], Thrombin Time [s], Quick [%], INR [dimensionless] and fibrinogen level measured via Clauss method [mg/dl]. *Right column:* pH values measured at respective time points

All results are given as median values. The Interquartile Range (IQR) is given in brackets

### Clot initiation and formation

**Fig. 1:**
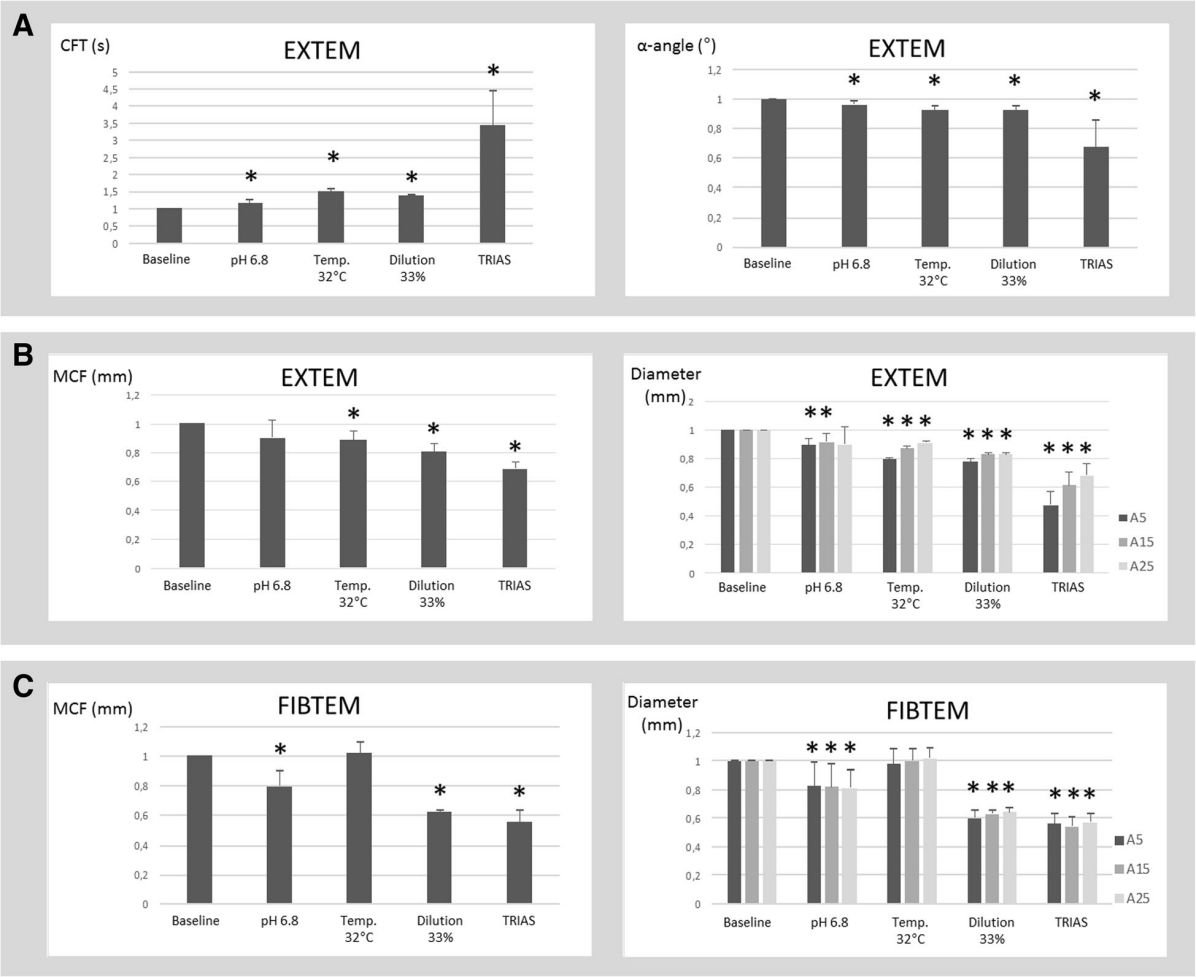
Clot kinetics and clot formation in ROTEM^®^. Functional coagulation testing using ROTEM^®^ analysis is presented as relative changes referring to the respective baseline coagulation. Baseline coagulation is set as 100%, respectively. Results are marked as statistically significant if their error probability was less than 5% (*p* < 0.05; *). **a**: Clot kinetics in EXTEM, specified by CFT and α-angle. **b:** Clot formation in EXTEM, specified by MCF and clot diameter after 5 min (A5), 15 min (A15) and 25 min (A25). **c:** Clot formation in FIBTEM, specified by MCF and clot diameter after 5 min (A5), 15 min (A15) and 25 min (A25)

EXTEM CTs in blood samples exposed to all three conditions remained unchanged as compared to baseline. In contrast, all conditions were associated with significant changes in EXTEM CFTs and α-angles, reflecting impaired clot dynamics (EXTEM CFT LT/WB = 3.42 s; *p* = 0.0008; EXTEM α-angle /WB = 0.67°; *p* = 0.004; Fig. 1a). This phenomenon was most pronounced when all three conditions were combined in the lethal triad preparation.

Assessing clot formation, EXTEM MCF decreased under the given conditions in the ranked order acidosis<hypothermia<dilution. Under lethal triad condition, the greatest impact on clot diameter was observed (EXTEM MCF LT/WB = 0.69 *p* < 0.0001). Interestingly, under conditions of lethal triad, we found a stronger impact on clot firmness for early time points (EXTEM A5) compared with late (EXTEM A25), resulting in a higher gradient of increasing clot size over time (c.f. Fig. 1b). In contrast, early FIBTEM time points (FIBTEM A5) were affected to the same extent compared to late (FIBTEM A25), without any significant relative clot assembly over time (Fig. 1c).

Accordingly, early and maximum FIBTEM amplitudes were significantly reduced under experimental acidosis, dilution and lethal triad, but not when exposed to isolated hypothermia. Exposure of blood samples to isolated acidosis was associated with a 20% decline in early and maximum FIBTEM clot amplitudes, while isolated dilution and combined lethal triad were associated with an almost 50% decline in early and maximum FIBTEM clot amplitudes, reflecting substantially impaired clot stability under the given conditions (Fig. 1c).

### Platelet contribution and functional platelet analysis

**Fig. 2:**
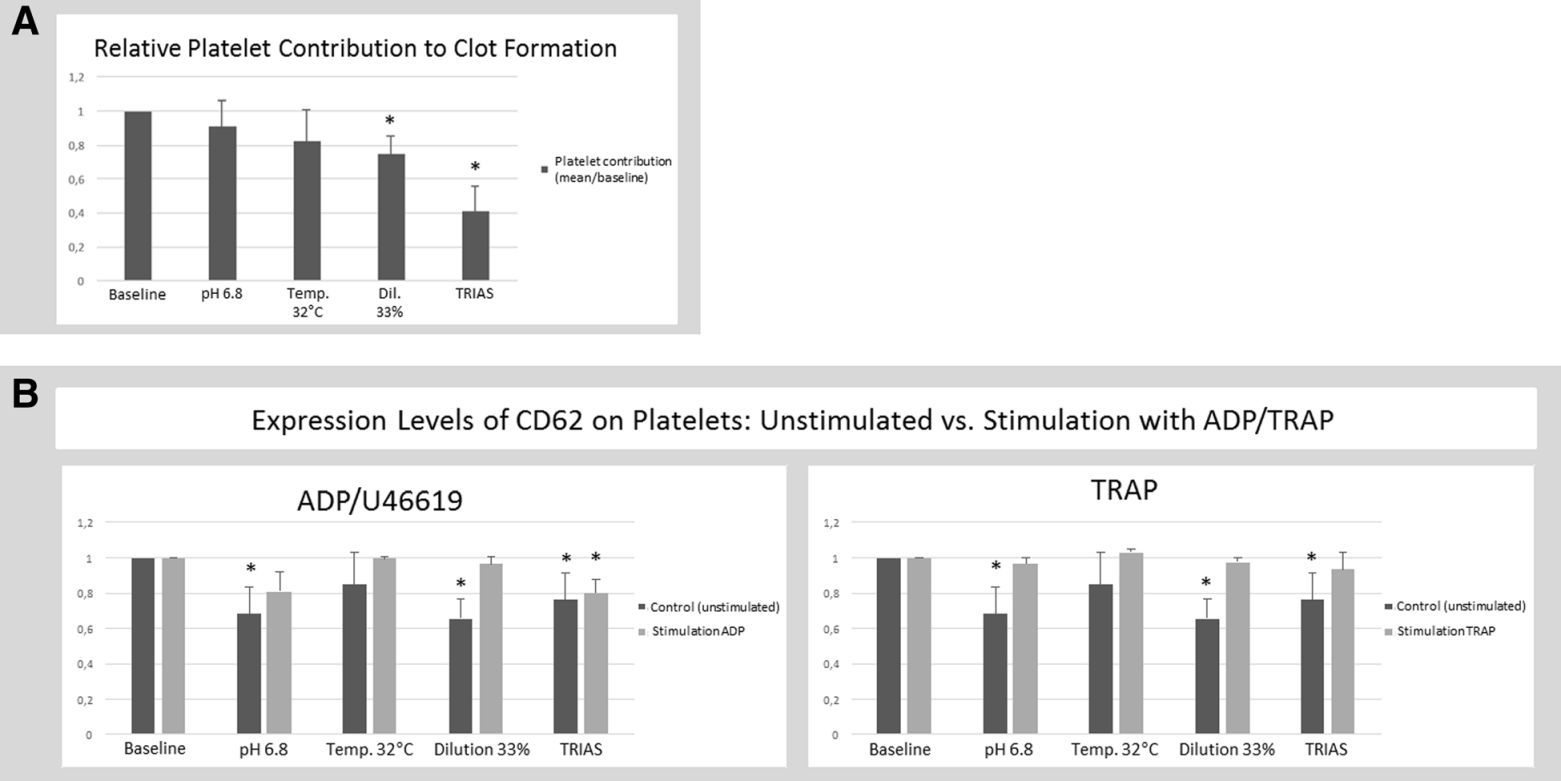
Platelet contribution under condition of lethal triad. **a:** Functional analysis of platelet contribution to clot formation using ROTEG PlateletMapping^®^. Results are presented as relative changes referring to the respective baseline (set as 100%). Results are marked as statistically significant if error probability was less than 5% (*p* < 0.05; *). **b:** Expression levels of CD62 on platelets surface are presented. Respectively, for each condition of lethal triad the expression levels unstimulated are shown (left column, c.f. “control, unstimulated”) and expression levels after stimulation (right column) with ADP/U46619 (left diagram) and with TRAP (right diagram). Results are presented as relative changes referring to the respective baseline (set as 100%). Results are marked as statistically significant if error probability was less than 5% (*p* < 0.05; *)

Platelet contribution to clot formation under the respective conditions was investigated comparing expression levels of CD62 on platelet surface at baseline and after stimulation with ADP or TRAP. Unstimulated expression gradually decreased under the given conditions, reaching statistical significance with experimental acidosis (Δacidosis/baseline 0.32; *p* = 0.006), dilution (Δdilution/baseline 0.34; *p* = 0.01) and combined lethal triad (Δlethal triad/baseline 0.24; *p* = 0.01, Fig. 2b). In addition, reduced response after stimulation with ADP under lethal triad condition could be observed reaching similar values compared to the acidosis condition but only reaching statistical significance (Fig. 2b). For all other conditions, in case of stimulation with ADP or TRAP no significant change could be observed (Fig. 2b). This effect was confirmed by investigating the functional platelet contribution to clot formation using ROTEG® platelet mapping. Platelet contribution decreased gradually under the respective conditions, reaching statistical significance for simulated dilution, and attaining its greatest extent under the conditions of lethal triad (Δtrias/baseline 0.59; *p* = 0.01, Fig. 2a).

## Discussion

The study aimed to elucidate the contribution of external factors — either isolated or combined in the context of the lethal triad — to the aggravating hypocoagulative state in TIC.

Based on the in vitro study design and a comprehensive coagulation analysis, interference of external triad factors in TIC and their impact on different pathways in coagulation were analysed.

### The lethal triad in TIC

To date, only a few studies have focused on the interference of external triad factors, and none has investigated the combination of all three in vitro [18]. Under all three conditions, we observed an additive effect resulting in significantly impaired clot formation and propagation, along with reduced clot firmness. Interestingly, initiation of clotting as reflected by clotting times was neither disturbed under isolated nor under combined conditions.

### The role of acidosis in TIC

Acidosis in trauma is mostly related to tissue hypoperfusion accompanied by an anaerobic metabolism, but may also result from resuscitation with chloride-containing fluids or blood stored in citrate phosphate dextrose adenine solution [19]. Acidosis has been shown to directly reduce coagulation capacity [20] via shifts in pH reaction optimum [15, 21, 22] and structural changes of coagulation factor Ca^2+^-binding sites [23]. A reduction in pH to 7.0 drops factor VIIa concentrations by > 90%, the FIIa/tissue factor complex by 55%, and the FXa/FVa complex by 70%, along with thrombin generation impairment [24]. In the present study, experimental acidosis was associated with thromboelastometric signs of impaired clot dynamics and stability. In a previous study, a pH reduction to 6.8 resulted in reduced clot stability, but also in prolonged clot initiation, which was not observed in the present study [25].

The most profound effect in the present study was observed in the FIBTEM assay, which reflects fibrin polymerization. As clot formation kinetics highly depend on the availability of fibrinogen and platelet function, it may be speculated that this effect was caused by fibrinogen dysfunction [18]. Experimental evidence suggests that acidosis increases fibrinogen breakdown by factor 1.8, with no impact on fibrinogen synthesis [26].

### The role of hypothermia in TIC

Considering mild hypothermia to be therapeutically effective for neuroprotection, the potential impact of hypothermia on haemostasis is of particular interest [10, 27, 28]. For a long time, coagulopathy recognized under hypothermic conditions was hypothesized to result mainly from reduced protease activity of clotting enzymes [29]. However, several in vivo studies could not verify this effect under mild hypothermia, and concluded that the overall enzymatic process in haemostasis is not significantly affected by a reduction from normothermia to 32 °C [30–32]. Furthermore, mild hypothermia does not seem to influence the clotting onset [33, 34]. These findings are consistent with our results obtained from standard coagulation assays, which essentially measure a sum of enzymatic steps, as well as from our viscoelastic test results assessing clotting times, which remained unaltered under the hypothermic conditions exposed in the present study. In contrast, clot dynamics in terms of CFTs and α-angles were significantly altered when blood samples were exposed to hypothermia, and thus confirmed previous in vitro studies revealing a significant correlation between temperature and haemostasis, with lower temperatures leading to an impairment of coagulation [11, 35]. Due to the fact that coagulation initiation reflects initial thrombin generation, and fibrin formation is mainly based on plasmatic proteases activity that is not impaired by hypothermia to such an extent as assumed, it may perhaps explain why only a slight tendency was observed for clot initiation times [36]. Considering clot stability parameters with the given reference ranges, fibrin polymerization may not, then, be affected by hypothermia.

### The role of dilution in TIC

In the clinical scenario, uncritical prehospital volume resuscitation has been identified as an independent risk factor for impaired coagulation profiles on admission [37]. In vitro studies revealed that dilution > 40% was required to induce coagulopathy [38], but retrospective registry data from trauma patients reported lower quantities as necessary to induce a bleeding phenotype with coagulopathy [16]. The experimental 33% dilution model used in the present study confirmed prolonged clot formation and propagation, as well as reduced clot firmness. The reduction in clot amplitude in the FIBTEM assay corresponded to reduced quantitative fibrinogen concentrations after dilution.

### Platelet dysfunction in TIC

Due to complexities in platelet testing, only a few studies have addressed platelet dysfunction in trauma and its contribution to TIC. However, near-total impairment of clot formation can occur as a result of platelet dysfunctions that are not detected by plasma-based coagulation testing [39]. The few studies that are available for platelet analysis in trauma use a variety of testing methods. To date, there has been no direct comparison of TEG-based platelet mapping with results from impedance aggregometry and direct measurement of CD62 expression using a flowcytometric assay. In this study, we used two different methods, but — quite apart from the fact that more research has to be done to elucidate the role of platelets in TIC — correlation of these different methods is of particular importance.

The data we derived from ROTEG^®^ platelet mapping indicate a significant impaired responsiveness with greatest impact for dilution and lethal triad. No impact on ADP stimulation was seen under the condition of hypothermia, which is in line with recent findings by Xavier et al., who describe enhanced platelet aggregation and ability to stimuli with ADP under conditions of hypothermia [40]. It can be assumed that impairment under conditions of lethal triad indicates a cumulative effect.

Using the flow-cytometric assay, under conditions of lethal triad, almost no stimulation with ADP was observed, resulting in a higher expression rate which indicated that the ADP pathway plays an important role in mediating platelet dysfunction under these external factors.

Comparing 51 trauma patients with 39 healthy controls, Wohlhauer et al. found evidence for significant reduced contribution to clot formation after stimulation with ADP within their trauma group, which is in accordance with our results. This reduced responsiveness to ADP has been attributed to a desensitization following continued exposure to ADP that is known to result from tissue injury and hypoperfusion [41, 42]. This might also be an effect in trauma, revealing an acquired defect due to circulation of exhausted platelets following prolonged activation. Otherwise, our data additionally provides evidence that hyporesponsiveness to ADP stimulation is also affected by external conditions as present under TIC.

A retrospective analysis of 163 trauma patients (mean ISS of 18) by Solomon et al. reported platelet hyporesponsivness to ADP and TRAP stimulation, and values below the normal range were significantly more frequent in non-survivors than in survivors (*p* = 0.0017 and *p* = 0.0002) [43].

Using a flow-cytometric assay to investigate ADP- and TRAP-mediated platelet aggregation response, Ramsey et al. found significantly lower levels of platelet activation and platelet function in 40 trauma patients (mean ISS of 10, IQR 2–18) compared to control subjects [44]. Based on the most significant reduction of TRAP, responsiveness in patients with head injuries, and the fact that TRAP served as a surrogate thrombin agonist, they suspect that thrombin-mediated activation of platelets via PAR-1 receptor may be restricted following an unknown pathomechanism. Whether this effect results from the particular entity of TBI, as some recent studies suggest, or could be attributed to a cross-link mechanism of platelet-coagulation within the trauma pathomechanism in TIC, has to be clarified in further studies [44, 45].

The present study has several limitations. In vitro settings are not able to fully reflect the pathophysiological mechanism present in trauma patients. Therefore, one should only cautiously extrapolate present findings to the setting presented in vivo or in a clinical situation. However, to isolate the respective conditions and to investigate the relevant interactions and their contribution to a resulting coagulopathy is not feasible in living human.

## Conclusion

Our study continues to support the concept of TIC as an endogenous entity comprising all the components of haemostasis, which follows rapidly on tissue injury, hypoperfusion and haemorrhagic shock. In addition, secondary factors of the lethal triad contribute to a different extent to a hypocoagulative state and have a direct impact on clot formation and platelet function as measured by thrombelastometry. Hence impairment of platelet function plays a key role in the pathophysiology of TIC. Thrombelastometric testing enables rapid evaluation of coagulopathy introduced by external factors after trauma, in contrast to standard coagulation testing. Our data indicates that conditions of lethal triad have an independent effect on platelet function, resulting in platelet dysfunction and reduced responsiveness to stimulation with ADP that might also be present after trauma. Therefore, impairment in primary haemostasis after major trauma should be taken more into consideration and included in the methodological setting of coagulation analysis after trauma.

## Abbreviations

aPTT: Activated Partial Thromboplastin Time
BGA: Blood gas analysis
CFT: Clot formation time
CPD: Citrate-phosphate-dextrose-derivate
CT: Coagulation time
MCF: Maximum clot firmness
POC: Point-of-care
TIC: Trauma-induced coagulopathy
TRAP: thrombin receptor activating peptide
